# Teaching Shared Decision Making to Family Medicine Residents: A Descriptive Study of a Web-Based Tutorial

**DOI:** 10.2196/mededu.6442

**Published:** 2016-12-19

**Authors:** Maxime Dion, Ndeye Thiab Diouf, Hubert Robitaille, Stéphane Turcotte, Rhéda Adekpedjou, Michel Labrecque, Michel Cauchon, France Légaré

**Affiliations:** ^1^Population Health and Practice-Changing Research GroupCHU de Québec Research CentreSaint-François-d'Assise HospitalQuebec, QCCanada; ^2^Department of Mathematics and StatisticsUniversité LavalQuebec, QCCanada; ^3^Department of Community HealthUniversité LavalQuebec, QCCanada; ^4^Department of Social and Preventive MedicineUniversité LavalQuebec, QCCanada; ^5^Department of Family Medicine and Emergency MedicineUniversité LavalQuebec, QCCanada

**Keywords:** decision making, patient participation, education, medical, graduate, educational measurement, program evaluation, computer-assisted instruction

## Abstract

**Background:**

DECISION+2, a Web-based tutorial, was designed to train family physicians in shared decision making (SDM) regarding the use of antibiotics for acute respiratory infections (ARIs). It is currently mandatory for second-year family medicine residents at Université Laval, Quebec, Canada. However, little is known about how such tutorials are used, their effect on knowledge scores, or how best to assess resident participation.

**Objective:**

The objective of our study was to describe the usage of this Web-based training platform by family medicine residents over time, evaluate its effect on their knowledge scores, and identify what kinds of data are needed for a more comprehensive analysis of usage and knowledge acquisition.

**Methods:**

We identified, collected, and analyzed all available data about participation in and current usage of the tutorial and its before-and-after 10-item knowledge test. Residents were separated into 3 log-in periods (2012-2013, 2013-2014, and 2014-2015) depending on the day of their first connection. We compared residents’ participation rates between entry periods (Cochran-Armitage test), assessed the mean rank of the difference in total scores and category scores between pre- and posttest (Wilcoxon signed-rank test), and compared frequencies of each. Subsequent to analyses, we identified types of data that would have provided a more complete picture of the usage of the program and its effect on knowledge scores.

**Results:**

The tutorial addresses 3 knowledge categories: diagnosing ARIs, treating ARIs, and SDM regarding the use of antibiotics for treating ARIs. From July 2012 to July 2015, all 387 second-year family medicine residents were eligible to take the Web-based tutorial. Out of the 387 eligible residents, 247 (63.8%) logged in at least once. Their participation rates varied between entry periods, most significantly between the 2012-2013 and 2013-2014 cohorts (*P*=.006). For the 109 out of 387 (28.2%) residents who completed the tutorial and both tests, total and category scores significantly improved between pre- and posttest (all *P* values <.001). However, the frequencies of those answering correctly on 2 of the 3 SDM questions did not increase significantly (*P*>.99, *P*=.25). Distribution of pre- or posttest total and category scores did not increase between entry periods (all *P* values >.1). Available data were inadequate for evaluating the associations between the tutorial and its impact on the residents’ scores and therefore could tell us little about its effect on increasing their knowledge.

**Conclusion:**

Residents’ use of this Web-based tutorial appeared to increase between entry periods following the changes to the SDM program, and the tutorial seemed less effective for increasing SDM knowledge scores than for diagnosis or treatment scores. However, our results also highlight the need to improve data availability before participation in Web-based SDM tutorials can be properly evaluated or knowledge scores improved.

## Introduction

Acute respiratory tract infections (ARIs) are the main cause of consultation in family medicine units in North America [1]. Despite numerous evidence-based guidelines [2-8] demonstrating that antibiotics are ineffective for treating most ARIs [9-12], primary care physicians seem unable to break the habit [13,14]. Widespread overuse of antibiotics for treating ARIs ultimately creates antibiotic resistance [15,16]. In a shared decision making (SDM) approach, health professionals explain the risks and benefits of the available treatment options to patients based on the best available scientific data and take into account patients’ values and preferences before making the treatment decision together [17,18]. Over the years, SDM has been recognized as an effective strategy for reducing the overuse of treatment options not clearly associated with benefits for patients [19]. Despite the willingness of policy makers in many industrialized countries to implement SDM in their health care systems, implementation has not been widespread in clinical practice [20], and few medical curricula include SDM training [21,22]. For this situation to change, SDM should be taught as early as possible in medical training and also as part of continuing education programs [23-25].

Web-based learning has become an increasingly popular approach to medical education [26,27] and is now ubiquitous in university education [28]. Although some have raised concerns about its effectiveness [27], Web-based learning modules have proved to be efficient in targeting many types of health professionals [26,29,30] for various purposes, including reducing the overuse of antibiotics. Little et al [30] recently conducted a study to assess the impact of a Web-based training intervention that aims to optimize the prescription of antibiotics for ARIs among general practitioners in 6 European countries. Their training program showed that Web-based training to enhance communication skills significantly contributed to a decrease in the prescription of antibiotics for treating ARIs. Although they did not focus specifically on SDM, there is widespread consensus that risk communication skills are one of its most important components [31]. Web-based programs have also been shown to be popular among residents. In 2005, Cook et al led a randomized controlled trial to find out whether there was a difference in internal medicine residents’ preferences between a Web-based module and printed materials and the level of knowledge achieved [26]. They found that the participants preferred the Web-based module because it saved time and concluded that Web-based learning was effective, well-accepted, and efficient [26].

In 2010, our team developed a multicomponent intervention (DECISION+2) for family medicine residents at Université Laval and for all health care professionals in the university’s family practice training units (FPTUs) [25,32]. Its final version contained a Web-based tutorial entitled “Shared decision making to treat ARI,” a 2-hour workshop in the form of a classroom course, and a decision aid. The impact of the full DECISION+2 was assessed as part of a cluster randomized trial that included 9 FPTUs and first- and second-year residents [33]. The results of this study showed that DECISION+2 contributed to reducing the number of patients deciding to use antibiotics for ARIs by facilitating their involvement in the treatment decision (to take antibiotics or not). The Web-based tutorial is currently mandatory for second-year family medicine residents at Université Laval, Quebec, Canada.

The potential of Web-based learning as an instructional tool for medical education has been recognized for many years [34,35]. However, little is known about how effective Web-based learning is for increasing SDM knowledge among physicians [36], how to evaluate usage and participation, and what kind of data are needed for these purposes. The objectives of this study were, therefore, to (1) describe the use of this Web-based training platform by the family medicine residents over time and its effect on knowledge scores (primary outcomes) and (2) note any gaps in data available for these purposes (secondary outcome).

## Methods

### The Web-Based Tutorial

**Figure 1:**
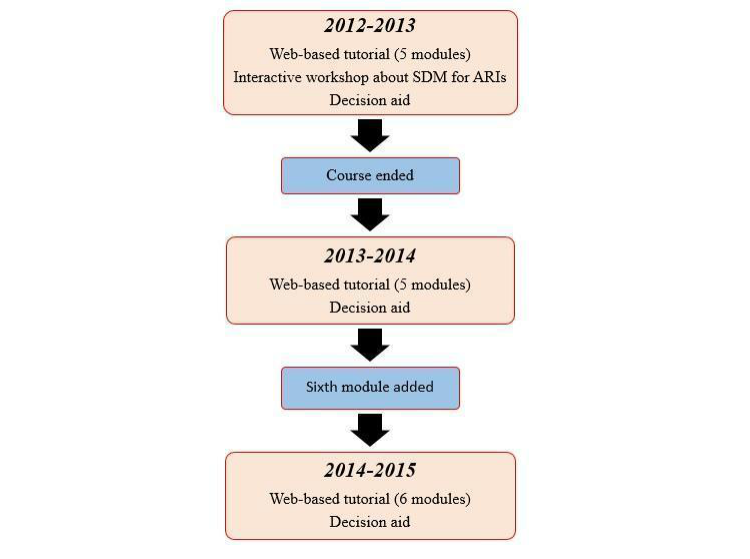
History of Université Laval SDM training program between July 2012 and July 2015. SDM: shared decision making; ARIs: acute respiratory tract infections.

The SDM training program has changed twice over 3 years. It was introduced in the family medicine residency program in 2011 as a multicomponent program: a Web-based tutorial entitled “Shared decision making to treat ARI,” a 2-hour workshop in the form of a classroom course, and a decision aid. However, the 2-hour workshop was withdrawn after the 2012-2013 residency period, leaving only the Web-based tutorial and the decision aid. The rationale for this removal was the time constraints in the residents’ schedule. Before July 2014, the Web-based tutorial contained 5 modules including information about diagnosis, treatment, and key components of the SDM process in the treatment of ARIs in primary care. After the 2013-2014 residency period, a sixth module on integrating the knowledge acquired in the first 5 modules was added. This addition also integrated parts of the workshop that had been removed 1 year earlier. Figure 1 shows a timeline of the major modifications made over the 3 residency periods.

The SDM tutorial was offered to all second-year residents in the entire network of 12 FPTUs of the Department of Family Medicine and Emergency Medicine at Université Laval, Quebec, Canada. This tutorial is one of the several tutorials available via the intranet on the department’s Web platform. At the beginning of their second year of residency, residents were given the link and invited to complete the tutorial and the pre-posttests over the course of the year. On the Department home page, residents entered their student identification number and password to access the tutorial. Then, they encountered a brief description of the tutorial before starting it (Textbox 1) [33]. The tutorial included videos, exercises, a link to access the decision aid, and a pre-post knowledge test. This tutorial was designed to be completed in 2-3 hours. It was required as part of the family medicine curriculum but not specifically evaluated. However, at the end of their residency, residents were examined on all the subjects they learned via the intranet.

**DECISION+2, a Web-based self-tutorial in shared decision making.**

Module 1: Introduction

- Introduce the shared decision-making process and acute respiratory infections

Module 2: Diagnostic probabilities

- Know the most useful signs and symptoms for the diagnosis of acute respiratory infections
- Integrate notions of diagnostic probabilities
- Know how to use diagnostic tools

Module 3: Treatment

- Know evidence on the effects of antibiotics in treating acute respiratory infections
- Integrate the concepts of probability associated with the effects of antibiotics in treating acute respiratory infections
- If the option for antibiotics is selected, choose which one

Module 4: Effective communication of risk and benefits

- Understand the essential elements of effective communication of treatment options and their benefits and risks
- Use the communication tool on the benefits and risks associated with using antibiotics or not to treat acute respiratory infections

Module 5: Promoting active patient participation

- Ask questions related to patient preferences and values, such as questions regarding their concerns about the benefits and risks associated with taking antibiotics or not
- Use a visual tool to help patients clarify their values and preferences about the benefits and risks associated with taking an antibiotic or not
- Verify patient comfort with the decision made

Module 6 (added after July 2014): Integrate all acquired knowledge

- Estimate diagnostic probabilities
- Effectively communicate the benefits and risks
- Identify the values and the preferences of the patient
- Promote an informed choice based on the best evidence available and that reflects what is important for the patient

### Participants

We included all second-year family medicine residents in the Department of Family Medicine and Emergency Medicine at Université Laval from July 2012 to July 2015 who logged in on the department’s Web platform to register for the tutorial, whether they completed it or not. Residency begins on July 15 of each year.

### Data Collection

To describe the participation in and usage of the DECISION+2 Web-based tutorial used to teach SDM at Université Laval and its effect on knowledge scores, we collected all available data about (1) its content and scoring system; (2) its history and the incentives offered for undertaking it; (3) residents’ participation in and usage of the tutorial; and (4) changes in their pre-post test scores. In observing the history of the SDM program, we noted any changes made to it and the reasons for change. Data for tutorial registrations between July 2012 and July 2015 were extracted from department’s Web platform. Data included identification number of each registrant, date of the first and last connection, frequency of connection, total time spent on the tutorial, and answers to each of the pretest and posttest questions. With the connection frequencies we were able to determine how many registered residents started the tutorial. Residents who logged in were separated into 3 entry periods (2012-2013, 2013-2014, and 2014-2015) depending on the day of their first connection to the tutorial. For example, a resident who logged in to the tutorial on March 23, 2013, was classified in the 2012-2013 cohort because he or she began between July 15, 2012, and July 14, 2013. We considered the tutorial as completed if the residents answered all the pretest and posttest questions.

The knowledge test used for the pretest and the posttest contained 10 multiple-choice questions and was only available in French (Université Laval is a French-language university). It was based on information in the tutorial and contained key elements considered by the authors as essential knowledge for the practice of SDM regarding the use of antibiotics for treating ARIs. Some questions were multiple response while others were single response, and participants did not know which kind they were answering. Four questions were concerned with diagnosis (all single response), 3 were concerned with treatment (2 single response and 1 multiple response), and 3 were concerned with SDM (1 single response and 2 multiple response; Multimedia Appendix 1). The knowledge scores were displayed as follows: for single response items, 1 point for a correct answer and 0 points for any incorrect answer; for multiple response items, 1 point if all answers were correct and 0 if any answer was missing or incorrect. The maximum score was 10 points. Residents could only see their scores at the end of the posttest questions.

### Statistical Analysis

We performed simple descriptive statistics including frequencies, median, and interquartile range (minimum and maximum) to summarize characteristics and modalities of use among all family medicine residents who logged in to the Web-based tutorial, and to understand how residents used the tutorial. Also, we estimated proportions of family medicine residents, per period, who logged in to the tutorial, did the pretest only, did the posttest only, or did both. The Cochran-Armitage test for trend was performed to test the change in proportions of family medicine residents who logged in over time. Because the knowledge score was an ordinal variable and did not respect the normality assumption, we used nonparametric tests. To describe the change in the level of knowledge among those who completed the tutorial, we used Wilcoxon signed-rank test to identify significant pre- or posttest differences between the total scores and scores on each of the 3 knowledge categories (diagnosis, treatment, SDM). This kind of test is used especially for paired samples. We used McNemar test to measure if residents answered the questions correctly after doing the tutorial. Finally, the distribution of knowledge total scores and category scores between periods were compared using Mann-Whitney U test. We considered a difference statistically significant when the *P* value was <.05. We performed statistical analysis using the SAS version 9.4 (SAS Institute Inc).

### Ethics

As the study was supported by the institution where it was performed, no ethical approval was requested because the Web-based tutorial was part of an academic program and data were provided anonymously.

## Results

### Participants’ Use of the Tutorial

**Figure 2:**
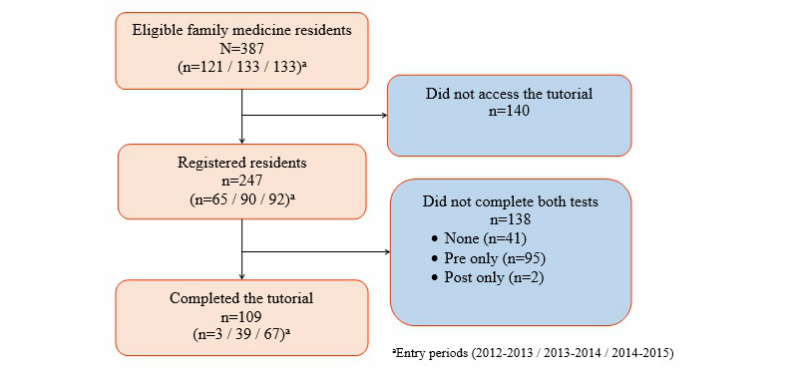
Flowchart of the participants.

All 387 second-year family medicine residents were eligible to take the Web-based tutorial. Out of the 387 residents, 247 (63.8%) logged in to the Web-based tutorial. Among the 247 who logged in, 109 (44.1%) completed both the pre- and posttest, 95 (38.5%) completed the pretest only, 2 (0.8%) completed the posttest only, and 41 (16.6%) logged in but did not complete either test. In total, only 28.2% (109/387) of all eligible family medicine residents completed the tutorial (Figure 2).

Table 1 shows simple descriptive statistics of participants for the different entry periods. Proportions of women and the median number of connections were similar between cohorts. However, a relative increase in the median of time spent in the tutorial was observed between the 2013-2014 and 2014-2015 cohorts. Also, the proportion of registered residents who logged in to the tutorial per entry period was 53.7% (65/121) in 2012-2013, 67.7% (90/133) in 2013-2014, and 69.2% (92/133) in 2014-2015 (Cochran-Armitage test; *P*=.006). Participation rates increased between each succeeding entry period and seemed more pronounced between the 2012-2013 and 2013-2014 cohorts.

**Table 1:** Description of the characteristics and modalities of use of family medicine residents who entered the tutorial.

Characteristics		2012-2013	2013-2014	2014-2015	Total
		n=65	n=90	n=92	N=247
**Gender, n (%)**					
	Female	54 (83)	69 (77)	70 (76)	193 (78)
	Male	11 (17)	21 (23)	22 (24)	54 (22)
**Number of connections**					
	Median	2	3	3	2
	IQR^a^	(1, 4)	(1, 5)	(2, 4)	(1, 4)
	Range	1-7	1-14	1-10	1-14
**Total time passed (hours)**					
	Median	1.54	1.87	2.77	2.22
	IQR^a^	(0.09, 2.71)	(0.49, 3.10)	(1.24, 3.87)	(0.38, 3.32)
	Range	0.00-10.13	0.00-9.29	0.00-19.98	0.00-19.98
**Tests done, n (%)**					
	None	18 (28)	15 (17)	8 (9)	41 (17)
	Pretest only	44 (68)	34 (38)	17 (18)	95 (38)
	Posttest only	0 (0)	2 (2)	0 (0)	2 (1)
	Pre- and posttest	3 (4)	39 (43)	67 (73)	109 (44)

^a^IR: Interquartile range (Q_1_, Q_3_).

### Family Medicine Residents’ Knowledge Scores

Twelve out of 109 residents who completed both tests had a posttest score equal to or lower than their pretest score, that is, the percentage of participants whose total knowledge score did not improve was 11%. Table 2 shows the medians and the interquartile ranges in the total knowledge scores and in each category among those who completed the tutorial. The median total knowledge score and each of the 3 category scores improved significantly between the pre- and posttest (Wilcoxon signed-rank test; all *P* values <.001). Table 3 shows the frequencies of those who answered each question correctly pre- and posttest. More participants answered the posttest questions correctly (McNemar test; all *P* values <.01) except for questions 7, 8, and 9 (the SDM category). In addition, low frequencies of those who answered correctly were observed for both pre- and posttest on questions 8 and 9.

**Table 2:** Medians and interquartile ranges in the knowledge total scores and for each category of those who completed the tutorial.

Category	Pretest, median (IQR^a^)	Posttest, median (IQR)	*P* value^b^
All (out of 10)	4 (3-5)	7 (6-8)	<.001
Diagnosis (out of 4)	2 (1-2)	3 (2-4)	<.001
Treatment (out of 3)	2 (1-2)	3 (2-3)	<.001
Shared decision making (out of 3)	1 (0-1)	1 (1-1)	<.001

^a^IQR: interquartile range.

^b^Difference assessed with Wilcoxon signed-rank test. *P* values do not represent the median difference but represent improvement in the mean rank of the difference in scores between the pre- and posttests.

**Table 3:** Frequencies of those who correctly answered each question.

Category		Pretest, n (%)	Posttest, n (%)	*P* value^a^
**Diagnosis**					
	Question 1	96 (88.1)	106 (97.2)	.008
	Question 2	14 (12.8)	64 (58.7)	<.001
	Question 3	27 (24.8)	68 (62.4)	<.001
	Question 4	41 (37.6)	90 (82.6)	<.001
**Treatment**					
	Question 5	40 (36.7)	101 (92.7)	<.001
	Question 6	36 (33.0)	90 (82.6)	<.001
	Question 7	100 (91.7)	103 (94.5)	.41
**Shared decision making**					
	Question 8	6 (5.5)	6 (5.5)	>.99
	Question 9	9 (8.3)	13 (11.9)	.25
	Question 10	66 (60.6)	92 (84.4)	<.001

^a^Tutorial effect assessed with McNemar test.

**Table 4:** Medians and interquartile ranges in the knowledge total scores and for each category between the 2013-2014 and 2014-2015 cohorts.

Knowledge test		2013-2014, median (IQR^a^)	2014-2015, median (IQR)	*P* value^b^
**Pretest**					
	All categories	4 (3, 5)	4 (3, 5)	.17
	Diagnosis	2 (1, 2)	1 (1, 2)	.96
	Treatment	1 (1, 2)	2 (1, 2)	.11
	SDM^c^	1 (0, 1)	1 (0, 1)	.69
**Posttest**					
	All categories	7 (6, 8)	7 (6, 8)	.95
	Diagnosis	3 (2, 4)	3 (3, 4)	.45
	Treatment	3 (3, 3)	3 (2, 3)	.36
	SDM	1 (1, 1)	1 (1, 1)	.98

^a^IQR: interquartile range.

^b^Distribution difference assessed with Mann-Whitney U test. *P* values do not represent the median difference but represent a distribution difference between the 2 entry periods.

^c^SDM: shared decision making.

Table 4 shows the medians and the interquartile ranges, and the *P* values of the 2-sided Wilcoxon rank-sum test performed to verify whether the distribution of total scores and the category scores pre- and posttest were the same between entry periods. The 2012-2013 period was not assessed because only 3 family medicine residents completed the tutorial during that period. All *P* values were greater than 5%, that is, there was no significant difference in the distribution of the total score or in any of the category scores for the pre- and posttests between the 2013-2014 and 2014-2015 cohorts.

Overall, we observed that the data available regarding residents’ participation in, use of, and effects of the Web-based tutorial on knowledge scores were limited. For example, sex was the only demographic data available, and data on time spent on the tutorial by residents per connection period, which pages they visited per connection period, and their participation in the classroom workshop were not available.

## Discussion

### Principal Findings

This study described the residents’ use of a Web-based training platform over time and attempted to assess whether the residents’ knowledge scores about the diagnosis and treatment of ARIs and SDM regarding the use of antibiotics for ARIs were improved by this Web-based training. It also provided an opportunity to identify what kinds of data are appropriate for evaluating the usage of the training platform and its impact on knowledge. The main results were that residents’ use of the Web-based tutorial increased over time, but not their knowledge scores; residents appeared to perform better on knowledge scores about diagnosing ARIs and treatment options than on SDM; just over a quarter completed the tutorial and one-third did not even start it; and little data appropriate for evaluating the course’s effectiveness were available. Our results led us to make 4 main observations.

First, we reported an increase in the participation rate between the 2012-2013 and 2013-2014 cohorts. This improvement could be explained by the removal of the workshop component of the course because 2012-2013 was the last year in which it was offered in addition to the Web-based tutorial. Perhaps family medicine residents saw the Web-based tutorial as an unnecessary addition to the classroom workshop on SDM in their curriculum and saw it as too much time spent on the topic. Some studies suggest that such Web-based tutorials should be brief and not too complex or intensive for medical students [24,37]. Furthermore, the average increase of about 1 hour in the median of the time spent in the tutorial observed between the 2013-2014 and 2014-2015 cohorts could have been caused by the addition of the sixth module. However, the added module was supposed to integrate all the knowledge acquired in the first 5 modules and compensate for the removal of the classroom workshop. We expected to see an increase in the distribution of the knowledge scores (total and per category) between these 2 periods, but this was not the case. Perhaps the inclusion of an additional review module was not relevant and may even have unnecessarily extended the duration of the training. More data would be needed to confirm this.

Second, our results suggested that the Web-based tutorial had a significantly positive effect on knowledge scores about the diagnosis and treatment of ARIs. However, the questions that the most residents failed were in the SDM category. This could be because the questions were poorly written, or it could be due to the nature of SDM. Unlike diagnosis or treatment, SDM is a subject that is inherently antithetical to unidirectional learning—it is about person-to-person communication and sharing information [17,18]. Perhaps Web-based tutorials performed in solitude are not an appropriate platform for teaching some of the essentials components of SDM to family medicine residents, namely presenting options, communicating risks and benefits, and clarifying values of patients. Moreover, knowledge scores may be an inadequate form of evaluation for SDM. However, in a 2013 randomized controlled trial on physician communication regarding prostate cancer screening, Feng et al assessed a 30-minute Web-based module and found, at 3-month follow-up, that the family physicians who used the Web-based tutorial had more shared decision-making behaviors and were more likely to encourage patients to consider different screening options compared with usual education [38]. Together, these results suggested that Web-based learning about SDM needs to be reassessed in further studies. They might also reflect the significant heterogeneity among SDM training programs [39], not only in their content but in their modes of delivery and evaluation of knowledge acquired. In this era of rapidly growing numbers of SDM training programs [40] and national efforts to offer them on the Web platform, the most effective methods of delivery and evaluation urgently need to be standardized.

Third, a difficulty we encountered was inadequacy of data organization or availability at the university level, which made it hard to evaluate the usage of the SDM training and its effects on knowledge. Indeed, the data available were not adequate for evaluating associations between participation in the tutorial and its impact on the residents’ knowledge scores. Moreover, no data were available that could inform us about whether the loss of the workshop component of the SDM program caused the increase in residents’ use of the tutorial. In addition, although all participants were second-year family medicine residents, we were unable to collect any demographic data on participants except for gender. If we had been able to analyze data extracted from log-in dates, visited pages, and time passed on each page per connection, our interpretation would have been more meaningful. To improve a Web-based training such as this, more information is needed about how the residents use the tutorial, and data collection needs to be adapted to reflect modifications in the program when they take place [41]. With the increase in Web-based interventions, the potential for data extraction is growing exponentially [42]. Moreover, sophisticated data analysis methods already exist that take account of the structure of more complex data such as this [43]. Our findings highlight the need to strengthen partnerships with residency programs so that data are made available in an appropriate form to be useful for evaluation purposes, both by faculties and by researchers.

### Limitations

The limitations of this study included contamination by residents who spent more than 1 year completing their second-year residency, and potential confounding variables. Lack of available demographic data compounded these problems. In terms of the tutorial’s effectiveness, participants were not classified by FPTU. Therefore, we cannot be sure that belonging to the same FPTU did not influence their answers. Also, participants might have logged in to the tutorial and then, rather than doing the tutorial, left the connection open and done something else for several hours (offline) before disconnecting. This might have distorted the time shown as spent on the tutorial. Finally, the psychometric properties of the pre- and post-knowledge tests had not been validated, and therefore scores might not have been valid, consistent, or reliable.

### Conclusions

Residents’ use of this Web-based tutorial appeared to increase between entry periods following the changes to the SDM program, and the tutorial seemed less effective in the SDM categories than in the diagnosis and treatment categories. However, to evaluate the use of a Web-based tutorial properly and its impact on knowledge, data collection needs to include the different log-in dates, visited pages, time passed on each page per connection, and more complete sociodemographic characteristics. There is still work to be done to improve data sharing, quality, and availability for evaluation purposes, so that implementation of SDM in the context of antibiotics use for treating ARIs becomes a feature of everyday family practice.

## Multimedia Appendix 1

Knowledge test.

## Abbreviations

ARI: acute respiratory tract infection
FPTU: family practice training unit
SDM: shared decision making
