# Supplementary material for: Teaching Shared Decision Making to Family Medicine Residents: A Descriptive Study of a Web-Based Tutorial
Source: JMIR Med Educ. 2016 Dec 19;2(2):e17. doi: 10.2196/mededu.6442 (PMC5206485; doi:10.2196/mededu.6442)
Supplement: Supplementary file 1 [file mededu_v2i2e17_app1.pdf]

Knowledge Test

The correct answers are underlined.

Q1. Which one of the following symptoms is the best indicator of a bacterial (vs. viral) sinusitis?

a. Unilateral sensitivity to pressure on maxillary sinus
b. Duration of symptoms
c. Greenish nasal secretions
d. Dental pain
e. Non-response to decongestants.

Q2. Out of 100 patients who have had a cough for less than three weeks, how many will have pneumonia if their vital signs and pulmonary exam are normal?

a. 0
b. 5
c. 10
d. 15
e. 20

Q3. Among these signs observed during an otoscopy exam, which one or ones would confirm the presence of a middle ear blockage?

a. Bulging of the typanic membrane
b. Erythema of the tympanic membrane
c. Absent or limited mobility in the tympanic membrane
d. Air-fluid level behind the tympanic membrane
e. Otorrhea

Q4. Out of 100 patients of 25 years old with a sore throat, with a temperature of 38.5, with no cough, with tender anterial cervical adenopathy and tonsillar exudates, how many will have Group A ß-hemolytic streptococcal pharyngotonsilitis?

a. 10
b. 25
c. 40
d. <u>55</u>
e. 70

Q5. Out of 100 patients who take antibiotics for acute rhinosinusitis, how many, on average, will get better or have their symptoms relieved one week later, due to the antibiotics?

a. <u>10</u>
b. 25
c. 40
d. 55
e. 70
f. 100

Q6. How many children with acute otitis media need to be treated with antibiotics to avoid one case of mastoiditis?

a. 108
b. 350
c. 489
d. 1005
e. <u>4064</u>
f. 110,043

Q7. Which of the following is the best choice for treating Group A ß-hemolytic streptococcal pharyngotonsilitis in an adult?

a. Amoxicillin 500 mg BID x 10 days

b. Azithromycin 500 mg DIE on 1st day then 250 DIE x 4 days

c. <u>Penicillin V 600 mg BID x 10 days</u>

d. Ciprofloxacin 500 mg DIE x 10 days

Q8. Which of following strategies is/are effective for communicating to the patient the risks and benefits associated with taking antibiotics for treating an acute respiratory infection?

a. <u>Using visual aids</u>

b. Avoiding use of numbers

c. <u>Explaining the scientific validity of the information</u>

d. Giving the relative risks of getting better with and without antibiotics

e. Putting more emphasis on the benefits than on the risks

f. <u>Indicating the probabilistic nature of the information.</u>

Q9 During a clinical encounter with a patient suffering from an acute respiratory infection (ARI) and for whom antibiotics is being considered, shared decision making is appropriate if (one or more answers possible):

a. <u>Evidence has shown that an antibiotic is more effective than a placebo for treating my patient.</u>

b. <u>Evidence has shown that taking antibiotics involves as many benefits as risks of side effects.</u>

c. <u>The quality of the evidence regarding antibiotics being effective for treating my patient is mediocre.</u>

d. <u>There is no available data that proves the effectiveness of antibiotics for treating my patient.</u>

Q10. To assess if a patient is comfortable with a decision about treating an ARI with antibiotics or not, which of the following considerations is optional?

a. Their understanding of the information about the risks and benefits of taking antibiotics or not.

b. <u>The decision support they can expect from other people.</u>

c. How sure they are about the decision.

d. The importance they assign to the benefits and risks of taking antibiotics or not, depending on their values and preferences.
